# Resistance to interprofessional collaboration in in-service training in primary health care^*^

**DOI:** 10.1590/1980-220X-REEUSP-2021-0473en

**Published:** 2022-05-13

**Authors:** Luana Pinho de Mesquita Lago, Daniel Vannucci Dóbie, Cinira Magali Fortun, Solange L’Abbate, Jaqueline Alcântara Marcelino da Silva, Silvia Matumoto

**Affiliations:** 1 Universidade de São Paulo, Faculdade de Odontologia de Ribeirão Preto, Departamento de Estomatologia, Saúde Coletiva e Odontologia Legal, Ribeirão Preto, SP, Brazil.; 2 Universidade Estadual de Campinas, Faculdade de Ciências Medicas, Departamento de Saúde Coletiva, Campinas, SP, Brazil.; 3 Universidade de São Paulo, Escola de Enfermagem de Ribeirão Preto, Departamento de Enfermagem Materno-infantil e Saúde Pública, Ribeirão Preto, SP, Brazil.; 4 Universidade Federal de São Carlos, Departamento de Enfermagem, São Carlos, SP, Brazil.

**Keywords:** Interprofessional Education, Internship, Nonmedical, Education, Public Health Professional, Institutional Practice, Primary Health Care, Qualitative Research, Educación Interprofesional, Internado no Médico, Educación en Salud Pública Profesional, Práctica Institucional, Atención Primaria de Salud, Investigación Cualitativa, Educação Interprofissional, Internato não médico, Educação Profissional em Saúde Pública, Prática Institucional, Atenção Primária à Saúde, Pesquisa Qualitativa

## Abstract

**Objective:**

to analyze the resistance to interprofessional collaboration in the professional practices of residents in primary health care.

**Method:**

Social and clinical qualitative research with 32 residents of a Multiprofessional Residency, carried out from 2017 to 2018. Data production included Institutional Analysis of Professional Practices, document analysis; investigator’s diary; and observation. Data were analyzed based on Institutional Analysis concepts.

**Results:**

There were contradictions between the reproduction of uniprofessional education with a focus on the specialty and interprofessional collaborative practices. The resistance analysis pointed to two axes: not-knowing as an analyzer of resistance to collaboration; interprofessional interference and knowledge-power relations. Residents’ practices were characterized as resistant to interprofessional collaboration.

**Conclusion:**

The resistance analysis in the Multiprofessional Residency showed integrative movements of assimilation and disputes with physician-centered power, with damage to the sharing of care and interprofessional communication. The collective analysis questioned health professionals education, revisiting the perspective of comprehensive care guided by the users’ needs.

## RESUMO

**Objetivo:**

fazer uma análise pelas resistências à colaboração interprofissional nas práticas profissionais de residentes na atenção primária à saúde.

**Método:**

Pesquisa qualitativa Sócio-clínica com 32 residentes de uma Residência Multiprofissional, realizada de 2017 a 2018. A produção de dados incluiu Análise Institucional das Práticas Profissionais, análise documental; diário do pesquisador; e observação. Os dados foram analisados a partir de conceitos da Análise Institucional.

**Resultados:**

Revelaram-se contradições entre a reprodução da educação uniprofissional com foco na especialidade e práticas colaborativas interprofissionais. A análise resistencial apontou dois eixos: não-saber como analisador de resistências à colaboração; interferências interprofissionais e relações de saber-poder. As práticas dos residentes foram caracterizadas pela resistência à colaboração interprofissional.

**Conclusão:**

A análise resistencial na Residência Multiprofissional evidenciou movimentos integrativos de assimilação e disputas com o poder médico-centrado, com prejuízos ao compartilhamento do cuidado e à comunicação interprofissional. A análise coletiva questionou a formação de profissionais de saúde, revisitando a perspectiva do cuidado integral orientado pelas necessidades dos usuários.

## RESUMEN

**Objetivo:**

Hacer un análisis por medio de las resistencias a la colaboración interprofesional en las prácticas profesionales de residentes médicos en la atención primaria a la salud.

**Método:**

Investigación cualitativa socio clínica con 32 residentes de una Residencia Multiprofesional, realizada entre 2017 y 2018. La producción de datos incluyó Análisis Institucional de las Prácticas Profesionales, análisis documental; apuntes diarios del investigador; y observación. Los datos fueron analizados a partir de conceptos del Análisis Institucional.

**Resultados:**

Se revelaron contradicciones entre la reproducción de la educación uniprofesional con énfasis en la especialidad y prácticas colaborativas interprofesionales. El análisis de la resistencia destacó dos ejes: el no saber cómo método de análisis de resistencias a la colaboración; interferencias interprofesionales y relaciones de saber y de poder. Las prácticas de los residentes fueron caracterizadas por la resistencia a la colaboración interprofesional.

**Conclusión:**

El análisis de resistencia en la Residencia Multiprofesional evidenció movimientos integrativos de asimilación y disputas con el poder médico centrado, con daños a la división del cuidado y a la comunicación interprofesional. El análisis colectivo cuestionó la formación de profesionales de salud, revisitando la perspectiva del cuidado integral orientado por las necesidades de los pacientes.

## INTRODUCTION

The health care model proposed by the Brazilian Public Health System (*SUS*) is centered on Primary Health Care (PHC) and aims to put the expanded clinic into practice. Its implementation takes place in the midst of disputes and diverse interests, in a process of permanent construction driven by established forces favoring maintenance and instituting forces of transformation of practices, care, and of health professionals’ training guided by the users’ needs, to guarantee the principles of universality, integrality, and equity^(1)^.

Interprofessional Health Education has been proposed by the World Health Organization (WHO)^(2)^ and encouraged by the Pan American Health Organization (PAHO)^(3)^ in the Americas region to improve person-centered care through interactive and shared learning among professional areas and with the community. In addition, in Brazil, the teamwork model has been present in health policies since the creation of the *SUS* and in training policies, which reinforces the potential for expanded health care, from the perspective of health needs, users’ participation, valuing autonomy and protagonism in their self-care and the humanization of practices based on the development of bonding relationships between team professionals and people cared for^(4)^.

This study aims at the interprofessional training in the Health Multiprofessional Residency (*RMS*), a graduate certificate program focused on in-service training and aimed at professional categories that make up the health areas, except medicine^(5)^. In the process of institutionalizing *RMS*, interprofessional training, supported by comprehensiveness and sharing of knowledge, finds resistance, as the fragmented logic of undergraduate education is still dominant, which encourages the primacy of uniprofessional work^(6)^, while constituting a space for Health Permanent Education, with disputes and agreements.

When we refer to the interprofessional practice in PHC, we consider the relationships, the context, and the teams’ work organization^(7)^. Professionals in health services have different interconnected ways of acting, such as: teamwork, collaboration, coordination and construction of interprofessional networks, which must be activated in a contingent way, articulated to their specificities^(8)^. In the context of this work, interprofessional collaboration is highlighted, a flexible practice that requires shared responsibility, a certain interdependence among professionals, clarity of roles, objectives and user-centered care^(7–9)^ which is materialized in the following dimensions: governance, shared objectives, formalization, and internalization^(10)^.

Organizations like WHO^(2)^ and PAHO^(3)^ understand collaboration as a tool to promote health, rationalize resources and reorient the person-centered care model with coordination in PHC, goals that have not yet been achieved in a global context. Thus, also in Brazil, the perspective of user-centeredness reiterates the importance of dialogue with the people cared for, their families and communities^(9)^, essential aspects for quality of care from the perspective of interprofessionality.

Interprofessionality can be approached as an instituting movement, part of the institutionalization movement of university education for health professionals. Based on Institutional Analysis (IA), the theoretical-methodological framework adopted in this research, an institution is understood as a set of norms and rules permanently transformed into a continuous process of contradictions, resulting from the dispute between moments. The moment of the instituted, or moment of universality in which the concept expresses all its positivity, where the established rationality is found (rules, social forms, and codes); the instituting, or moment of particularity is presented as a negation of the preceding moment, there are events, developments, and social movements that question these norms. These conflicting forces generate a third movement-moment resulting from the dialectical contradiction between the two previous moments: the institutionalization process or singularity moment, which represents what we see concretely^(11)^.

The university education of health professionals, as an institution, carries its standards and is crossed by the instituted model of competitive education, which values individuality in learning at work and does not encourage a collaborative culture in health services. At the same time, there may be instituting forces in favor of the integration and sharing of knowledge- power, focused on collaboration. In these movements, there are resistances marked by the dispute of forces and the fragile integration among the different areas of knowledge and practices.

From the perspective of IA, resistance is defined as “a social force that updates itself in opposition to another (social force), called power. This contest of forces favors, at least provisionally, the latter^(12)^. Thus, resistance is produced and expressed in practices, in the functioning of establishments, and in the interface with other institutions present, such as: the technical and social division of work, professions, health, among others. Resistance analysis, or analysis *by* resistances, shall be distinguished from the analysis *of* resistances, since acts of resistance support the analysis and do not exist in isolation. Resistances are part of the social context that produces them and, as such, are traces of force that open paths for the subjects’ analytical work. Resistance as an analyzer can, therefore, reveal institutional contradictions, and even more, it can activate them, awaken them^(12,13)^.

Resistance is a dialectical concept didactically divided into three moments: defensive, offensive, and integrative^(12)^. The first is conservative, the second revolutionary, and the third is an alternative to this mutual opposition, of an adaptive character. They relate to and influence each other, and these relationships vary depending on the situation^(12)^.

In a work carried out in the health area based on this perspective, a group of mental health professionals reflected on the relevance of interprofessional and network work to sustain and produce comprehensive health care. The group gained legitimacy at the management by building a work group with more autonomy and the use of tools that brought them closer to self-management^(13)^.

This article’s objective is the analysis by resistances to interprofessional collaboration in the professional practices of residents in PHC. This becomes relevant considering that the interprofessionality movements in a Multiprofessional Residency Program and its repercussions on the PHC of the municipal network show some paths and challenges to bring out the potential for transforming practices that can be provoked by the resistances present in the in-service training.

## METHOD

### Design of Study

This is a research-intervention investigation with a qualitative approach, which portrays the results of a doctoral dissertation, in which concepts from the IA framework were adopted in the Institutional Socio-Clinical approach, theoretical-methodological framework for long interventions and the use of various devices, elaborated in the 2000s from a reinterpretation of social and analytical interventions^(14)^.

By refusing the traditional neutrality of the investigator and the distance between him/her and the research object, the Institutional Socio-clinic proposes the immersion of investigators in the field for the analysis of effects, in a work that reunites eight characteristics: analysis of the order and demand; participation of subjects in the approach under variable modalities; analyzers’ work giving access to questions that are not normally expressed; analysis of the transformations taking place as the work progresses; application of restitution modalities that return the provisional results of the work to the participants; work on primary and secondary implications; intention to produce knowledge; and attention to institutional contexts and interferences^(14)^.

### Population

The study was produced from July 2017 to February 2018 with residents from different areas of health: Pharmacy, Physiotherapy, Speech Therapy, Nutrition and Metabolism, Dentistry, Psychology, and Occupational Therapy.

### Local

The study was carried out in the city of Ribeirão Preto, SP, Brazil, in the Multiprofessional Residency Program in Comprehensive Health Care at the Medical School of Ribeirão Preto, Universidade de São Paulo.

### Data Collection

Data production was carried out by this article’s first author, a doctoral student at the time of the research, through devices such as document analysis, observation in Family Health Units (*USF*), Institutional Analysis of Professional Practices (AIPP), and support from the research journal.

The documental analysis included the analysis of the course’s pedagogical project and the call notice for residents. In the observation, the investigator followed moments of the practice of groups of three to seven residents in six *USF* from October to December 2017, and recorded in the research diary. The average observation time was two to three hours per week in each team, using an observation guide of the work dynamics and participants’ interaction, in interprofessional activities such as shared consultations, health promotion groups, team meetings, and matrix support meetings.

The AIPP, one of the modalities of the Institutional Socio-clinic^(14)^, was developed in eight monthly sessions, in a room at the University, in the format of conversation circles, lasting approximately one hour, which were audio-recorded and transcribed. The AIPP sessions took place in the common training space called Common Theoretical Module (*MTC*), from August 2017 to February 2018, with an average participation of 25 residents between R1 and R2, not including health professionals, preceptors or users, and there was a session with the participation of two professors invited by the residents^(15)^. Some examples of topics addressed in the *MTC* by residents were related to the Expanded Center of Family Health, Intersectoriality, and National Policy on Primary Care.

The sessions were conducted by the first author, who was close to the Institutional Socio-clinic and did not know the group members until the beginning of the study. From the records in the research diary, the investigator wrote a narrative and read it at the beginning of each session to trigger self- analysis movements in the group^(15)^.

### Data Analysis and Treatment

The research diary was used as a tool for recording during observations, in the sessions of analysis of professional practices and for the analysis of implications of the first author throughout the investigation process. The notion of institutional implication concerns the “set of relationships that exist, consciously or not, between the actor and the institutional system”^(11)^, between the investigator and the participants and each actor in relation to the institutions involving them. They can be affective, organizational, or ideological^(16)^.

Documentary analysis focused on historical basis^(17)^ that considered the conditions of document production, its use and dissemination, and the theoretical references on which they are based. This analysis allowed characterizing the course and movements of its institutionalization process.

At the end of the AIPP sessions, the corpus was organized and the data analyzed, supported by the theoretical- methodological framework of Institutional Analysis with an Institutional Socio-clinical approach, elucidating the main analyzers, that is, phenomena that revealed what was hidden in the institutions, and effects of research, that is, recurrent phenomena that were reproduced in the institutions^(18)^.

The transcribed material was read and the results obtained from the different devices were cross-referenced: practice analysis sessions, observations in the health units, document analysis, and research diary. The synthesis of the main results was presented to the participants in restitution sessions, a characteristic moment in socio-clinical research, carried out with the teams at the *USF* so that they could get to know and problematize the elaborated analyses. Sessions took place in January 2018, with the use of *Power Point* and a proposal for a play-role game for approximately one hour, on a date previously agreed with the proposition of a reflective moment on collaborative teamwork and records in the research diary.

### Ethical Aspects

The invitation to participate in the research was sent by email to all residents of the 2016–2018 (R2) and 2017–2019 (R1) classes and a meeting was scheduled to clarify objectives and formalize participation, by signing the Free and Informed ConsentForm (FICF). This study complies with Resolution 466/12 and was approved by the Research Ethics Committee under opinion number 2.111.731 in 2017.

## RESULTS

Thirty-two residents from different health areas participated in the study. There were 14 R2: Pharmacy (2), Physiotherapy (2), Speech Therapy (2), Nutrition (2), Dentistry (3), Psychology (1), and Occupational Therapy (2), and 18 R1: Pharmacy (3), Physiotherapy (2), Speech Therapy (3), Nutrition (2), Dentistry (3), Psychology (2), and Occupational Therapy (3).

The Multiprofessional Residency Program in Comprehensive Health Care provided a workload of 60% in PHC, and defined as the objective promoting teamwork focused on chronic non- communicable diseases in the municipal network. However, when analyzing the institutionalization process of this course, institutional interference was observed, represented by greater investments in research activities, and a centralized and productivist university management model that weakened collaborative pedagogical processes and created limits to comprehensive care in PHC. From the perspective of the Institutional Socio-clinic, it can be stated that there was an effect of falsification of the objective proposed at the time of its foundation, of its initial preview of comprehensive health care. The “Mühlmann Effect”, as it is also known, is a very common effect, characterized by a change of direction during the institutionalization process, motivated by bureaucratization. It is the moment when the institution betrays and falsifies the initial preview on which it was founded, evidencing the institutional failure^(18)^.

The results, presented below, analyze the dispute for power in the context of education and work in two axes of analysis: non-knowledge as an analyzer of resistance to collaboration; interprofessional interference and knowledge-power relations. Thus, they evidence the need to institute tools that could favor the sharing of roles and objectives, establish greater trust among them, and question the loss of user-centered care.

### Not-Knowing as an Analyzer of Resistance to Collaboration

In this axis, doubts about the different roles, professional competences and possibilities of interprofessional action had resistance to collaboration as an effect. Faced with their “not knowing”, the residents were self-isolated in their practices and, at the same time, brought into analysis the need for spaces to exchange information and discuss collaboration as tools to enhance problem-solving capacity in PHC. In the AIPP sessions, the residents analyzed their work with the other professionals of the teams and how much both resisted to learn to work together, which distanced them from the focus of integrality, favoring the instituted of the specialty.

During observation of the PHC teams and MTC throughout the AIPP, residents expressed the naturalization of the practice of “passing the case” to different specialists and the logic of medical-centered care, evidencing internal conflicts regarding the co-responsibility of care with the user: *I think we are reproducing the medical model a little bit because having 1,000 specialties so… it’s not an orthopedist… it’s the hand orthopedist… it’s the head and neck doctor* [ ] *So actually we (residents) end up reproducing these things…*[ ] *but sometimes he (user) would not need it, there are things that could be handled there (at the Family Health Unit) but it is a whole health system, of TRAINING in health…* (MTC – Occupational Therapy Resident R1 (A) 08/29/17).

In the previous report, the resident reflected on the limits of the problem-solving issue in PHC and the great expectation of the team for specialized individual care, a model instituted to the new specialist professional. The difficulty for residents to have an active voice in the team, compared to the other actors in the residency program, was a reflection of established hierarchical relationships and of not recognizing themselves as team members. However, dealing with these difficulties and lack of knowledge required a reflective monitoring related to the team’s work process and, sometimes, it was possible to talk about their responsibility in the search for filling the knowledge gaps: *I, particularly… I think the gaps (of knowledge) are not just within us… but in the coordination itself…* (MTC – Speech Therapy Resident R1 (B) 11/07/17).

In this perspective, the AIPP meetings were also permeated by a lot of silence, because the individualism present in their practices, sometimes, prevented them from exposing their non-knowledge and putting them under analysis in the group. Not-knowing, as an institutional production, was resignified, opening possibilities for the integration of professional practices. Self-analysis in the group of residents about their “knowledge gaps” revealed unsaid things, there was recognition of their not- knowing and that there are other people who also do not know, such as tutors and preceptors who are co-responsible for the teaching-learning process. The analysis of its implications from the place of training and the analysis by resistance to collaboration questioned the reproduction of the teaching method established at the University, characterized by the transmission of knowledge and hierarchy in the classroom, between those who know and those who do not know, and led to reflection on the protagonism and co-management movements in its training process.

By identifying their difficulties, institutional contradictions and blocking points, the group of residents set themselves to find solutions and expand communication channels with professors, inviting them to discuss the objectives of the *MTC* in training. In this process of recognizing its lack of knowledge and the challenges of its formation, the group managed, moderately, to democratize knowledge, power and manage decisions regarding the *MTC* together with the other actors in the residence.

### Interprofessional Interference and Knowledge-Power Relations

Knowledge-power relations, revealed after the interprofessional meeting and interprofessional interference, were present as resistances at the moment when institutions, intrinsic to the different professions, came up against, crossed and disputed with each other, bringing tension to the production of interprofessional collaborative care. In these interferences, the organizational and ideological implications provoked resistance to a more integrated model of work: *the difficulty in implementing this idea (of shared care) is actually how we organize ourselves so that the doctor can stay with me at the same time because of his schedule.* (MTC – Speech Therapy Resident R1 (A) 08/29/2017)

Collaborating with others does not only depend on an effort to make the user better understood, it is a process of human relations. These relationships permeate the organization of the work agenda and are crossed by a work management model based on the production of health as a quantitative machine. Many elements are added up when defining the agenda, such as choosing who will participate, how and for how long. Some teams tried to institute an integrated agenda for shared care between multiprofessional residents and physicians, but, unfortunately, the institutional dynamics maintained individual schedules and fragmented care.

Interprofessional interference determined the approach or distance of multiprofessional residents from medical professionals (both physicians from the unit and resident physicians). Some physicians were more open to dialogue in informal moments, such as a coffee break in the kitchen, recognizing these spaces as favorable for collaboration.

Even so, it was possible to observe, from the perspective of gender relations, gaps of affective implication in favor of collaboration produced in coexistence and in the construction of trust, for instance, in the case of a shared care among the female Speech Therapist, Physiotherapist and resident Physician (who also had a degree in Physiotherapy). In this case, the interprofessional relationship of trust among resident women made collaboration possible. The women’s resistance led to a confrontation with the established knowledge-power relationship, creating alternatives to the doctor-centered power instituted from an institutional gender interference.

Women were free to work together. They established a zone of trust with each other, with sharing of knowledge between female residents and the female user. For instance, a shared care was carried out with a female user, a young teenager, who was being treated for a facial paralysis. The collaborative practice included dialogue about the treatment, there was interaction about what would be the best decision in the resolution and care for the young woman, who was ashamed to smile, and the writing of information and observations in the medical record supported the matrix support. During treatment, the young woman expressed that she was happy with the improvement in her condition.

The female encounter acted as resistance in favor of collaborative care, as sharing flowed, produced partnership and dilution of knowledge-power. The meeting between the female physician, female physiotherapist and female speech therapist with the female user opened a breach, which made the practice of interprofessionality go beyond the established medical power. As a complement, it was important to restore the damaged aesthetic, making other implications of an affective character among women arouse, with the common objective of protecting the young woman’s appearance and defending her autonomy.

In another team, on the other hand, the matrix support meeting was an integration movement surrounded by institutional interference and resistance between medical and non-medical power. The preceptor female physician proposed a matrix support meeting, including medical residents, medical students, and multiprofessional residents. The space specifically accommodated medical care demands, while the other residents, who were available with their specialized knowledge, acted as paramedics, or assistants to the physicians.

In this case, the resident accommodated him/herself in the established knowledge-power relationship and remained integrated into a common project or objective, the doctor-centered model, without moving in search of integrality. This took place in such a way that this paramedic position or paramedical matrix support turned into resistance to interprofessional work.

## DISCUSSION

The results show different effects on the residents’ professional practices. The AIPP device, supported by the notes in the research diary and the narratives read in the group, addressed the analysis of orders and demands, the elucidation of analyzers, and the analysis by the resistances of the group^(15)^, mainly the confrontation between resistance to collaborative interprofessional practices and the established power of fragmentation and hierarchy among professions.

First, it is worth highlighting the effect of falsification of the initial preview, or “Mühlmann Effect”^(18)^, since, at the beginning of the study, the residents’ practices went in the opposite direction to what was provided for at the time of creation of the course, making them distant from the integrality of care and from their role as protagonists. At the same time, the residents, integrated into the established power, maintained the reproduction of the institution, reinforcing the non-integration of practices and the care that was not very user-centered, but more focused on fragmented professional actions.

Second, based on the “Lapassade Effect”^(18)^, residents found ways to resist institutional control mechanisms and create different arrangements in their training. The “Lapassade Effect” characterizes a social tendency to deviate from institutional rules in favor of the collective, with the invention of modes of operation that tend to resist the mechanisms of institutional control and domination^(19)^. A movement present in this process of institutionalization of in-service training included the organization of the agenda and the demand for pedagogical support for the investigator and course management.

The self-analysis movements, produced in the group of residents, allowed the appearance of a movement of getting free from blame from failure in the face of their not-knowing and a movement of re-accountability^(19)^, that is, the evidence of a certain autonomy of the group stemming from the analysis of orders and demands.

Scheduling a meeting with professors and coordination led to the expansion of communication and dialogue channels, which may have contributed, for a certain period, to the governance of interprofessional practices through connectivity among the different actors^(10)^, an important element of collaboration. From this perspective, residents approached co-management processes based on the sharing of knowledge and power^(20)^, but with no institutional guidance. Faced with the challenges arising from democratic institutional spaces, there is a range of devices that guide practices in the direction of co-management, with the common objective of creating dialogic environments within the workers, managers, and users in which problems, needs, and possible solutions are placed under analysis^(21)^.

In this study, there was a tendency to reproduce already known practices, when teams that lived under insecurity of the *common act* in the PHC were closed in themselves, maintaining walls between them. Thus, when referring to interprofessional collaborative practice, authors defend a contingency approach to teams, and the encouragement of a shared identity and responsibility, clear roles and objectives, interdependence, integration in team tasks^(7,8)^, aspects that were fragile in the institutional context studied, such as fragmented actions in health services, with few moments for case discussion or reflection on the work process within multidisciplinary teams.

The helplessness residents felt in the face of responsibility for their own training revealed an institutional functioning that surrendered to managerial accountability, the one produced by the demand of managers for performance from the public service and its actors^(22)^. This is a presupposition of the New Public Management paradigm or managerialism, which caused damage to the sharing of responsibilities in the urser’s care, and the strengthening of the logic of productivism.

In addition, the AIPP highlighted the valorization of the traditional method of university teaching based on the transmission of knowledge and the conflict in the face of the priority given by the university to scientific production based on performance and productivity^(23)^. This scenario reveals aspects of institutional policy, which gives little encouragement to teacher training and the implementation of new teaching methods in the training of health professionals towards more collaborative practices. This fact reiterates the importance of adopting processes of collective analysis, potentiating spaces for field tutoring, so as to allow finding new paths in multiprofessional residencies and moving towards a training model based on interprofessional health education that aims to strengthen the interprofessional collaboration.

In this regard, the professional implications^(16)^ indicated movements in favor and others against interprofessional practice. On the other hand, the affective implications favored the integration of practices, were related to the willingness to work together and to the experiences based on mutual trust and coexistence, the establishment of a bond with the user and with co-workers, which brought them closer to what is called internalization of collaboration^(10)^. In addition, the analysis of their common goals of user-centered care were reflected in the negotiation of common time in the agenda (organizational implication) and in the interest in solving a shared case (ideological implication with comprehensive care). Thus, the analysis of implications points out some paths for interprofessional collaboration.

As a limitation of this study, the gap in the analysis of users and health professionals about collaborative practices is considered, as they did not participate directly in the different moments of the study. Moreover, another dimension of interprofessional collaboration that needs to be strengthened in this context is governance, which includes, in addition to management guidance, aspects such as sharing of responsibilities with local leaders, continuing education processes, and maintenance of spaces favoring connection and the dialogue between its actors to discuss problems and solutions together^(10)^.

From this perspective, the recognition and creation of sharing and connectivity tools pointed to the direction of interprofessional collaboration in the daily life of PHC; however, the gaps mentioned above weakened its governance and formalization^(10)^. Despite this, the residents’ experience revealed tensions in the face of self-management, non-knowledge, knowledge-power relationships, challenges posed to the integration of practices and the achievement of comprehensive care centered on the user, guided by their health needs.

## CONCLUSION

Resistance in the residents’ professional practice clearly pointed to integrative movements of assimilation of physician-centered power, such as the positioning of residents as paramedics. However, this direction presents tensions and conflicts that shall be analyzed to face this established power, when the goal is achieving greater integration of practices, interprofessional collaboration and integrality in health care with a reduction of asymmetries and the search for balance in power relations.

Resistance to collaboration revealed knowledge-power relationships and disputes with medical power in care, with damage to the sharing of care and represent obstacles in interprofessional communication, establishment of partnership and interdependence. In view of the analysis of its implications, the user gained prominence when professionals recognized care as a common objective, even working interdependently.

Based on reflexivity, residents resignified their practices, taking positions and sharing decision-making spaces to qualify comprehensive health care. The need to expand the moments of co-management and reflection on common spaces for training and practices is evident, considering the power of these spaces to question their role as health professionals, and to resume their central objective of producing life, with emphasis on interprofessional collaboration focused on users’ health needs.

## ASSOCIATE EDITOR

Divane de Vargas

## > Financial support

Coordenação de Aperfeiçoamento de Pessoal de Nível Superior, doctoral scholarship social demand of the Postgraduate Program in Public Health Nursing (EERP-USP). “The present work was carried out with the support of the Coordenação de Aperfeiçoamento de Pessoal de Nível Superior – Brazil (CAPES) – Financing Code 001”.
